# Movement Detection in Soft Robotic Gripper Using Sinusoidally Embedded Fiber Optic Sensor

**DOI:** 10.3390/s20051312

**Published:** 2020-02-28

**Authors:** Mei Yang, Qidi Liu, Hamza Sayed Naqawe, Mable P. Fok

**Affiliations:** Lightwave and Microwave Photonics Laboratory, College of Engineering, University of Georgia, Athens, GA 30602, USA; meiyang@uga.edu (M.Y.); qidi.liu@uga.edu (Q.L.);

**Keywords:** soft robotic, movement detection, fiber sensor, fiber Bragg grating (FBG), stretchable sensor

## Abstract

Soft robotics is an emerging field, since it offers distinct opportunities in areas where conventional rigid robots are not a feasible solution. However, due to the complex motions of soft robots and the stretchable nature of soft building materials, conventional electronic and fiber optic sensors cannot be used in soft robots, thus, hindering the soft robots’ ability to sense and respond to their surroundings. Fiber Bragg grating (FBG)-based sensors are very popular among various fiber optic sensors, but their stiff nature makes it challenging to be used in soft robotics. In this study, a soft robotic gripper with a sinusoidally embedded stretchable FBG-based fiber optic sensor is demonstrated. Unlike a straight FBG embedding configuration, this unique sinusoidal configuration prevents sensor dislocation, supports stretchability and improves sensitivity by seven times when compared to a straight configuration. Furthermore, the sinusoidally embedded FBG facilitates the detection of various movements and events occurring at the soft robotic gripper, such as (de)actuation, object holding and external perturbation. The combination of a soft robot and stretchable fiber optic sensor is a novel approach to enable a soft robot to sense and response to its surroundings, as well as to provide its operation status to the controller.

## 1. Introduction

Soft robots are made from soft, elastic materials, and are capable of mimicking the complex motions of human and animals. Soft robots have attracted considerable attention [1], since they offer unique opportunities in areas where conventional rigid robots are not a viable solution. Materials with good stretchability can be used for building soft robots, with examples including shape memory alloys (SMAs) [2,3,4], shape memory polymers (SMPs) [5], electro-active polymers [6], acrylic [7] and silicone elastomers (i.e., polydimethylsiloxane (PDMS) [8] as well as silicone gel [4,9,10]). Soft robots that mimic the movement of animals have been explored, including a robot finger [8], soft cylindrical manipulator [11], soft gripper [12,13,14], starfish [4] and octopus tentacle [9]. Applications of soft robots for locomotion [4], bending and shape detection [11], movement detection [12], as well as temperature and strain sensing [8], have been demonstrated.

Embedded soft sensors are an essential component for soft robots to sense and respond to their surroundings, as well as provide status feedback to the controller, so that they can perform delicate and sensitive tasks. Stretchable electronic sensors, including the use of liquid metal, printable conductors, piezoresistive rubber and braided conductive wires [6,15,16,17], have been developed to overcome the stretchability and deformability challenges for sensing in soft robotics. Although existing stretchable electronic sensors have great success in robotic sensing, these approaches are based on variation in resistance, conductance and capacitance induced by the change in physical phenomenon; therefore, these stretchable electronic sensors cannot survive harsh environments that could be corrosive, explosive or under the effect of a strong electromagnetic field. 

Alternatively, fiber-optic based sensors have a number of unique properties that make them attractive for sensing in soft robots [18,19,20,21,22]. In particular, their light-weight, low-loss, fast response, electromagnetic interference immunity, chemical stability and nontoxic nature make them an ideal sensor in biomedical and harsh environments. However, the actuation of soft robots often leads to the elongation and continuum deformation of the robots—essentially inhibiting the use of conventional polymer or glass fiber optic sensors in soft robotics due to their stiffness and poor stretchability, as well as the difference of Young’s modules compared with the soft material used in soft robots. Research has shown that the use of a strain-limiting layer such as fabric can reduce elongation in soft robots [23]; however, delamination is still observed between the optical fiber sensor and the soft material if elongation has not been completely eliminated. A few stretchable fiber optic sensors were designed based on a light-guiding mechanism in a waveguide [24,25]; however, none of them were actually built using a conventional glass optical fiber, meaning that they do not carry all the advantages of using optical fiber as a sensor. For instance, a larger loss is observed in polymer fiber or special waveguide, even when the sensor signal only needs to be transmitted over a short distance of a few meters, thus limiting its applications where a longer distance transmission is required. Furthermore, the relatively large diameter in polymer optical fiber makes it hard to be embedded inside the thin wall of soft pneumatic robots. 

Fiber Bragg grating (FBG) is one of the most popular fiber optics sensors that has a mature fabrication process [8], and is sensitive to a number of physical phenomena and chemical parameters. Different kinds of FBG are designed and demonstrated for temperature, strain, bending, curvature, stretching and torsion sensing. Soft sensors based on FBG have also been used for strain and temperature sensing [8], shape reconstruction [18,26,27,28] and curvature measurements [20,29]; however, they are essentially non-stretchable due to the stiffness of glass optical fiber. Previously, we demonstrated a fiber optic stretchable sensor by embedding an FBG in a sinusoidal configuration at an off-set position in a silicone film [30,31]. The demonstrated sensor allows 30% elongation, and can sense tension, curvature and bending direction, as well as torsion direction. However, the study only focused on single static parameter sensing; continuous monitoring dynamic movements and events happening in a soft robot using the stretchable fiber optic sensors have not been proposed or studied.

In this paper, we demonstrate a pneumatic soft robotic gripper with an FBG sinusoidally embedded to one of its arms. This proposed work investigates the effect of sensor embedding location and angle, as well as the ability to monitor and detect continuous movements and events occurring at the soft robot, which is an important study to enable the practical implementation of using fiber optic sensors in soft robotics, and to provide essential feedback information to the soft robot controller. The unique sinusoidal configuration enables high sensitivity and repeatable movement detection in a stretchable soft robot, as well as facilitating accurate and fast response sensing. We experimentally investigated and compared the performance of both sinusoidal FBG configuration and straight FBG configuration under various conditions, including (de)actuating gripper, picking up objects of different weight and experiencing external perturbation on the object.

## 2. Fabrication of the Sensor and Operation Principle

### 2.1. FBG Embedded Soft Robotic Sensor Fabrication

**Figure 1:**
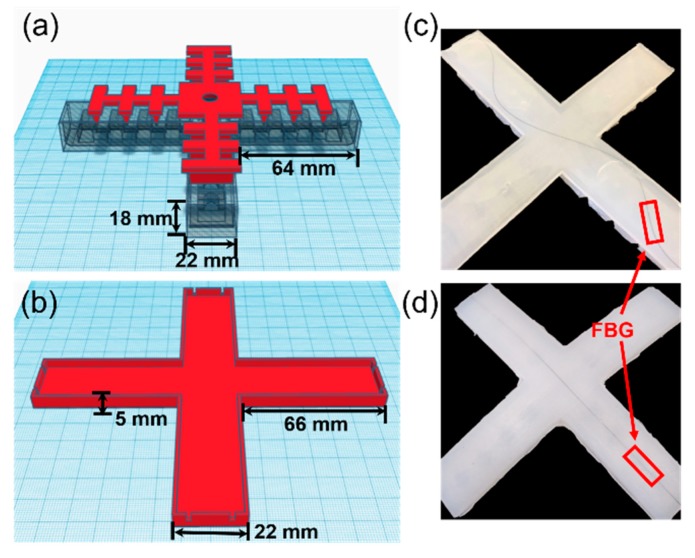
(**a**) 3D molds for fabricating the top part of the soft gripper; (**b**) 3D molds for fabricating the gripper base; (**c**) soft gripper with sinusoidally embedded FBG sensors; and (**d**) soft robotic gripper with straightly embedded FBG sensors. FBGs are indicated by the red box.

To fabricate the soft robotic gripper with a FBG sensor, a 3D printing technique is used to make the four molds, including the top layer of the gripper fingers with separators to ensure the fingers bend inwards (red in Figure 1a), air chambers (gray in Figure 1a) as the main node of inflation, the base of the gripper where the FBG sensor is embedded (Figure 1b) and the connector for connecting the air pump. In the gripper base mold, grooves are designed to hold the FBG in position when the silicone gel is curing. The size of each gripper finger is 64 mm in length and 22 mm in width. Two types of silicone gels (i.e., 00–10 for soft and 00–30 for hard) from Ecoflex are used to construct the soft robotic gripper. 

First, silicone gel 00–10 is poured into the first mold (Figure 1a) to create the air chamber, which helps the gripper to bend easily. The harder silicone gel, 00–30, is applied to the second mold (Figure 1b) to form a 1-mm thick film. Then, a 10-mm long FBG (Technica Optical Components, LLC) written in a standard single mode fiber (SMF) is placed on the 1-mm silicone film with either a sinusoidal or straight configuration. The two ends of the FBG are fixed at the grooves on the base, while 00–30 silicone gel is poured into the base mold to form another 1-mm thick silicone film for covering the FBG, resulting in a 2-mm thick silicone layer. When both the gripper top and base are cured, a thin layer of 00–30 mixture is used to attach the base to the gripper top. The curing of silicone gel is performed under the condition of 60 °C for 30 min. 

The FBGs (indicated by the red boxes in Figure 1c,d) used in the soft gripper have a center wavelength of around 1547 nm, 3-dB bandwidth of 0.25 ± 0.05 nm and a reflectivity of >92%. The arm length direction is the x-axis, and the arm width direction is the y-axis. The embedding sinusoidal shape is governed by the function y = sin(ωx), with ω = 1 and y = 1 cm, corresponding to an arm width of 2 cm. Therefore, the resultant spatial period of the sinusoidal shape is T = 2π/ω, which is 6.3 cm. The FBG fiber is embedded sinusoidally in the gripper arm at 1/4 position from the tip of the gripper arms for optimized sensing performance. The investigation of embedding position and period of the sinusoidal shape will be discussed in Section 3.

In our first model, a strain-limiting fabric is attached to the bottom of the gripper in attempt to eliminate elongation and delamination; however, our experiment shows that delamination between the straightly embedded optical fiber sensor and the soft material can still be observed, meaning that the strain-limiting fabric can reduce elongation but not completely eliminate it. Due to this, we decided to remove the strain-limiting fabric to simplify the fabrication process and reduce the materials needed for a more robust soft gripper. Furthermore, with the use of a sinusoidally configured fiber optic sensor, there is no need for a strain-limiting fabric to prevent elongation, while at the same time enabling movement sensing in the soft robotic gripper.

### 2.2. Sensing System

**Figure 2:**
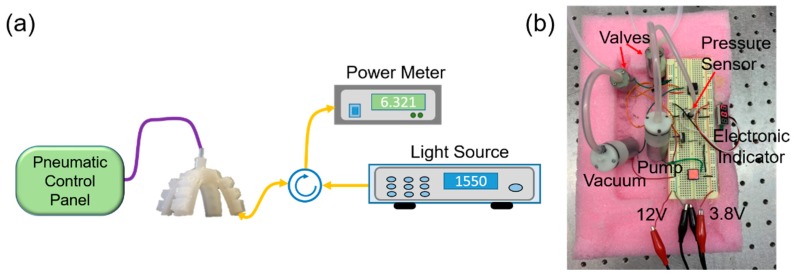
(**a**) Experimental setup for testing the FBG embedded soft robotic gripper. Yellow lines: optical paths. Purple line: air tube; and (**b**) picture of the pneumatic control panel.

Figure 2a shows the experiment setup of the FBG embedded soft gripper. Continuous wave (CW) light from the light source is launched into the FBG via an optical circulator, and is then reflected from the FBG and directed to the optical power meter (1830-R, Newport Corporation) or optical spectrum analyzer (OSA) (AP2040A, APEX Technologies) for spectral analysis. Optical paths are indicated by the yellow lines, while the air tube is denoted in purple. The gripper is connected with a pneumatic control panel using an air tube. An electronic pressure sensor in the control panel is used to monitor the pressure inside the gripper, as well as to keep the pumping strength the same for all actuations of the gripper.

Figure 2b shows the hardware of the designed pneumatic control panel that is powered by 12V DC and a 3.8V DC (tunable) power supplies. The control panel consists of two valves, a pump, a vacuum and an electronic pressure sensor. Air is trapped in the gripper when the valves are in the off state. When the pump is enabled, the soft robotic gripper is actuated, while deactuation of the gripper is carried out by enabling the vacuum. Due to the internal structure of the air chamber, the gripper fingers will be bent inward when air is pumped into the air chamber (actuation). When air is released from the gripper (deactuation), the gripper is deflated and returned to the natural bending state. 

### 2.3. Sensing Principle

**Figure 3:**
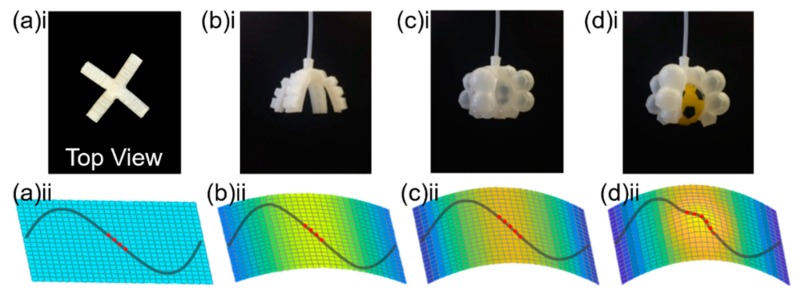
Soft robotic gripper at different operation states. **(a)i** Top view of gripper resting on a flat surface; **(a)ii** strain distribution in the soft gripper arm, where the gray solid curve is the optical fiber and the dashed red line is the position of the FBG; (**b**) natural bend; (**c**) gripper actuated without holding an object; and (**d**) the gripper actuated and holding a sphere.

In this part, we investigated the effect of various movements in the gripper on the FBG reflection spectrum, including when gripper is rested on a flat surface (Figure 3(a)i), the gripper is suspended to a natural bend position (Figure 3(b)i), the gripper is actuated without holding an object (Figure 3(c)i) and the gripper is actuated and holding an object (Figure 3(d)i).

Initially, the gripper is resting on a flat surface, and the optical fiber is embedded sinusoidally (the gray curve in Figure 3(a)ii, with the FBG region indicated by a dashed red line). When the gripper is suspended to a naturally bending position, the gripper fingers are slightly curved, which essentially bends the FBG, as illustrated in Figure 3(b)ii. The background color of the grids shows the strain distribution on the base of the gripper arm. Next, when the gripper is actuated through air-pumping (without holding an object), the gripper arms bend more and stretch, as shown in Figure 3(c)ii. When the gripper is actuated and holding a 60 mm sphere object, the object presses against the base of the gripper, inducing an additional outward force to the FBG; the base of the gripper is thus deformed, as illustrated in Figure 3(d)ii. 

As shown in Figure 3(b)ii and (c)ii, the FBG is folded, and experiences a positive strain when the gripper is actuated, resulting in an increment in the grating period (Λ). The relationship between the grating period and FBG Bragg wavelength $\lambda_{B}$ can be described as

(1) $$\lambda_{B}=2n_{eff}\cdot \Lambda$$

where $n_{eff}$ denotes the effective refractive index of the FBG. Therefore, as the gripper is actuated, from Figure 3a–d, the FBG Bragg wavelength will shift to the longer wavelength.

## 3. Experiment Results and Discussion

**Figure 4:**
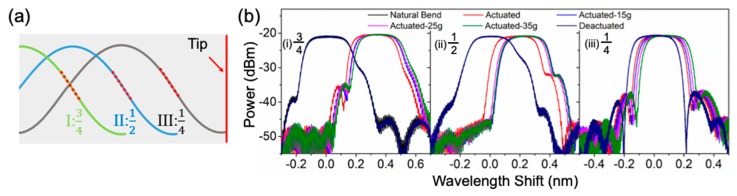
(**a**) Illustration of various FBG embedding positions in the gripper arm (red dotted lines indicate the location of the FBG); (**b**) effect on FBG wavelength shift in optical spectra due to different events occurring at the soft gripper (i) Position I: FBG embedded at 3/4 arm length from the tip, (ii) Position II: FBG embedded at 1/2 arm length from the tip, and (iii) Position III: FBG embedded at 1/4 arm length from the tip.

First, we investigated the optimized position to embed the FBG in the soft gripper arm. As illustrated in Figure 4a, three FBG positions are studied where the center of the FBG is at 3/4 (position I), 1/2 (position II) and 1/4 (position III) of the arm’s length away from the arm tip. The spectra of six different states at the soft gripper, including natural bend, actuated without holding an object, actuated and holding a 30-mm diameter sphere with weight of 15 g, 25 g and 35 g, as well as deactuation, are recorded as shown in Figure 4b, to identify the optimized embedding position. Using the natural bend state as the baseline, the measured wavelength shift is summarized in Table 1.

As observed, both position I and II have a significant wavelength shift when the gripper is actuated, but an insignificant change in wavelength shift is observed during the object weight change. Since the FBG is embedded at the upper positions of the gripper arm (3/4 and 1/2 from the tip), it is more sensitive to bending in the arm, but less sensitive to the object being held. On the other hand, position III has the FBG embedded at 1/4 from the tip. It shows a balance between its sensitivity to arm bending and the change in object weight, resulting in a more evenly distributed and significant wavelength shift. The uniform and noticeable wavelength shift allows a better differentiation between various status with either the wavelength or power measurement. The reflection wavelength does go back to the value at natural bending state after deactuation, meaning that no delamination is observed in any of the three cases. Therefore, FBG will be embedded at the 1/4 position in the gripper arm for the rest of the experiment for optimized sensitivity.

**Figure 5:**
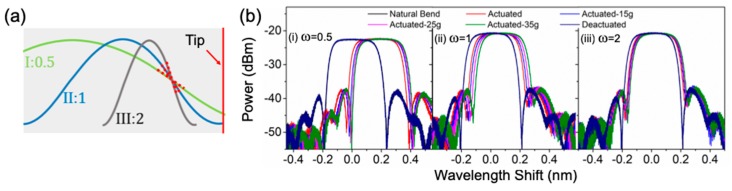
(**a**) Illustration of various FBG embedding sinusoidal period in the gripper arm (red dotted lines indicate the location of the FBG); (**b**) measured optical spectra of FBG corresponding to different events occurring at the soft gripper (i) Period I: ω = 0.5, (ii) Period II: ω = 1, and (iii) Period III: ω = 2.

Next, with the FBG embedded at the 1/4 position, we study the effect of the period of the sinusoidal embedding configuration, i.e., the FBG embedding angle, on the reflection wavelength shift. The spatial period of the sinusoidal shape (T = 2π/ω) is changed by setting ω to 0.5, 1 and 2, as shown in Figure 5a. The spectra of six different states at the soft gripper are measured, as shown in Figure 5b, and the measured wavelength shifts are summarized in Table 2. With ω = 0.5 (region I), a relatively large wavelength shift is observed when the gripper is actuated, but a small wavelength shift is resulted during the object weight change. When ω = 2 (region III), the wavelength shift is extremely small for all states, as the FBG is close to perpendicular with the bending, which makes it insensitive to any deformation in the gripper arm. For ω = 1 (region II), evenly distributed and significant wavelength shifts are observed, which allow a good differentiation between different states with either the wavelength or power measurement. Therefore, a 1/4 position and a ω = 1 period are used for embedding the FBG sensor in the soft robotic gripper in the experiment below. 

Although embedding the FBG at 1/4 position and using ω = 1 as the sinusoidal period provide the optimized wavelength shift, the amount of wavelength shift is still small, and a high-resolution OSA is required to detect the shift. To solve this challenge, optical power measurement is used to provide a more significant change in the measurement parameter, and reduce the cost of the system. Therefore, a CW laser and an optical power meter is used in place of the broadband light source and the OSA. A laser at 1549.96-nm and 1549.75-nm, corresponding to the plateau edge of their reflected spectrum at natural bending state, are chosen for the reflection power change measurement of the FBGs in sinusoidal and straight configurations to achieve maximum power change, respectively. The choice of wavelength is governed by the optimized performance of the FBG during the whole process, such that maximum power change could be obtained throughout the possible wavelength shift region of the embedded FBG.

**Figure 6:**
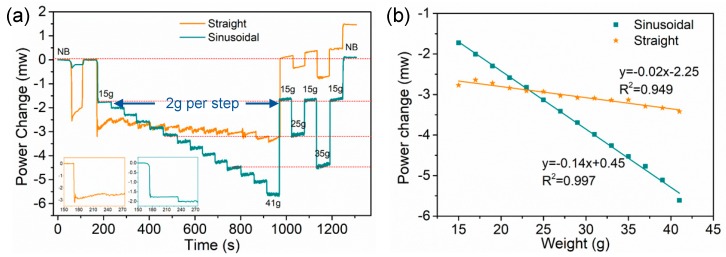
(**a**) The effect of weight change in the object to the reflected power change at the operation wavelength of the embedded (sinusoidal and straight) FBG in the soft gripper; (**b**) relationship between optical power change and object weight.

Furthermore, we compared the performance of the grippers with sinusoidally and straightly embedded FBGs for holding objects with various weights from 15–41 g. Power change is recorded using LabView at a 2-Hz sampling rate, as shown in Figure 6a. Constant air pressure is used to actuate the soft gripper. The turquoise curve is the measured result from the soft gripper with a sinusoidally configured FBG, while the orange curve is the measured result from the gripper with FBG in a straight configuration. Initially, the soft gripper is in a natural bend state (NB), though it is then actuated. In the gripper with sinusoidally configured FBG, the optical power is dropped by 0.34 mW during actuation, and is stabilized at a −0.20 mW position. Meanwhile, in the straight configuration, the optical power first drops down to −2.55 mW during actuation, is unable to be stabilized, and keeps increasing. The gripper is deactuated to NB, and is actuated again to hold a sphere with a weight of between 15–41 g. Insets in Figure 6a are the zoom-in view of the measured power change over time due to actuation. Power in the sinusoidal configuration changes immediately when the soft gripper is being actuated, and is stabilized instantaneously. However, the stabilization time the straight configuration gripper takes after weight changing is more than 60 s, i.e., power keeps increasing and cannot maintain a constant value. When the weight of the sphere is increased (at 230 s), power in the sinusoidal configuration changed instantaneously, while the straight configuration takes at least 2.5 s to respond to the weight changing. The instability and slow response time in the straight configuration are due to the delamination of FBG, which results in the relieving of tension over time.

Then, the weight of the object increases at a step of 2 g, with a 60 s interval in between. As shown in Figure 6a, the power measured in the straight configuration (orange curve) keeps increasing even when the weight is not changed, and a small increment in power change is also observed between each change in object weight. By contrast, the sinusoidal configuration (turquoise curve) has a clear and linear change in optical power, resulting from the object weight change from 15 g to 41 g, with 2 g per step. The relationship between the power change and the object weight is shown in Figure 6b. As shown by the turquoise curve in Figure 6b, the use of power measurement approach provides a high sensitivity and linear response. Specifically, it shows a linear fitting function of y = −0.14x + 0.45, with a fitting coefficient R^2^ of 0.997. The calculated weight sensitivity of soft gripper with a sinusoidally configured FBG is 0.14 mW/g. Similarly, we did the same measurement using a gripper with a straightly configured FBG. In this scenario, the total power change is less than 0.8 mW, resulting in a linear weight sensitivity of only 0.02 mW/g.

Next, we test the repeatability of the measurement by changing the weight of the object in the following sequence: 41 g, 15 g, 25 g, 15 g, 35 g and 15 g, and then deactuate to natural bending. The gripper with sinusoidally configured FBG has repeatable results (i.e., the power change values are the same as the earlier measurement of the same weight), indicated by the red dashed lines in Figure 6a. When the gripper is being actuated for a long time, a small increase in power is observed at each step of the weight change, which is due to a small air leakage in the pneumatic system. It shows that the embedded sensor is capable of monitoring any air leakage in the pneumatic system. The leakage problem can be mitigated using a better sealing at each connection. The straightly configured FBG, on the other hand, does not have repeatable results—the optical power bounced around the natural bending state due to the delamination of the FBG. Lastly, the gripper is deactuated and restored to its natural bending position. Only the gripper with a sinusoidally configured FBG can return to the natural bending value immediately.

**Figure 7:**
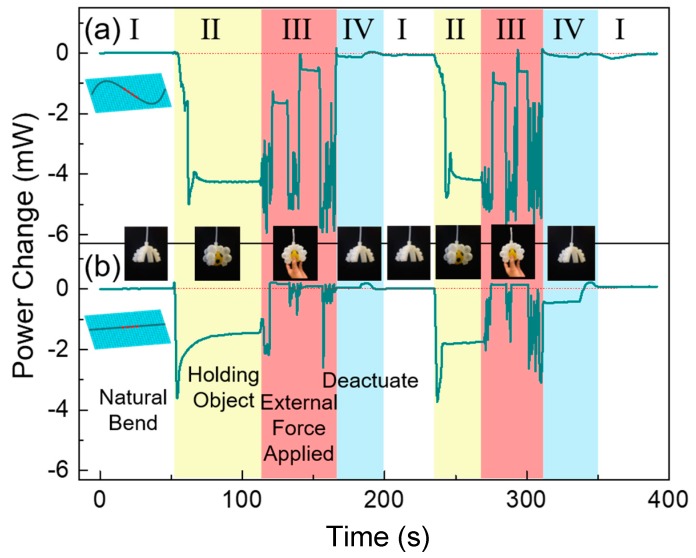
Power change measurement during various motions and events occurring at the soft robotic gripper: (**a**) with sinusoidally configured FBG; and **(b**) with straightly configured FBG.

In this part, both sinusoidally and straightly configured FBGs are compared for detecting various movements and events occurring at the soft gripper: actuating (with/without holding an object); under external perturbations (pulling/wiggling the object); and deactuating. The power change measurements of the two configurations at the operation wavelength are shown in Figure 7a,b. The gripper at the natural bending state (region I) is actuated to pick up an object. Once the gripper has a firm grip of the object (region II), an external force is applied to it, and attempts to pull and wiggle the object, resulting in the noise-like power change (region III). The external force attempted to pull the object out of the gripper without success, mimicking the object as stuck while trying to be picked up. When the external force is removed, the gripper returned to a steady state, as represented by the plateaus in region III. This process is repeated two times with an increased external force, where the object is finally removed. Then, the gripper is deactivated and is returned to the natural bending state after a small change in power (region IV). All of the above movements are repeated again to demonstrate their repeatability. The actuation and deactuation processes have been repeated over 50 times, and the power always comes back to the value of the natural bending state, which proves that no delamination is observed.

Comparing the power change of the sinusoidal and straight embedding configurations, the sinusoidal configuration has more distinguished features; the noise-like feature in the sinusoidal configuration (Figure 7a) is more consistent and significant than the straight configuration (Figure 7b). Furthermore, when the external force is removed, the power change will be stabilized at a value between the state of “holding an object” and “natural bending”, indicated by the plateaus in region III, with the exact power of this point depending on the force applied to the object, i.e., how much the object position has been moved inside of the gripper arm. The third plateau at the end of region III, consistent with that of region I (actuated without holding an object), reveals that the object is missing. However, in the soft gripper with straightly configured FBG, the power returns back to its natural bending state after the first pull action, with the gripper still being actuated and holding the object, giving false information about its status. The gripper with straightly configured FBG is not capable of differentiating various movements and events happening in the gripper, while the soft gripper with sinusoidally configured FBG provides useful and reliable information. From Figure 7, it is illustrated that the various movements and statuses of the soft robotic gripper can be clearly distinguished with the sinusoidally configured FBG by considering the pattern and amount of power change. This observation is summarized in Table 3. It is worth noticing that since our approach uses the magnitude and pattern of power changes to determine the corresponding event happening in the soft robotic gripper, it is more tolerable to laser power fluctuation. Fluctuation in laser power may result in a small amount of noise in the power change magnitude, but the overall trend should remain the same.

The performance of the soft robotic grippers with sinusoidally and straightly configured FBGs can be summarized by Table 4. A soft gripper with sinusoidally configured FBG has a higher sensitivity of 0.14 mW/g, while the gripper with straightly configured FBG only has a sensitivity of 0.02 mW/g. Delamination occurs in the soft robotic gripper with straightly configured FBG, resulting in poor repeatability, a slow response time and a slow stabilization time of the sensing and detection processes. Therefore, the sinusoidally configured FBG overcomes the intrinsic limitation in conventional glass optical fiber, making it a promising candidate for embedding in soft robots due to its stretchability, along with its advantages of being highly repeatable and fast responding, as well as having high sensitivity.

## 4. Conclusions

This paper presents the first demonstration of an air-actuated soft robotic gripper with sinusoidally embedded FBG for sensing the events and movements of the soft gripper. The sinusoidally embedding configuration has a number of unique advantages over straight embedding configurations, making it possible for optical fiber sensors to be used in soft robots. The embedded FBG unfolds with the arm as the soft gripper is actuated, preventing delamination from occurring, and resulting in a sensitivity that is seven times greater than its straight configuration counterpart, as well as good sensing stability and good sensing repeatability. Furthermore, the prevention of delamination in sinusoidal configuration also leads to a fast sensing response time, which is particularly important for movement detection in the gripper. Various movements, including (de)actuation, object holding and external perturbations, are clearly distinguished using the proposed soft robotic gripper with the unique FBG sensor embedding configuration.

## Figures and Tables

**Table 1:** Wavelength shift of FBG embedded in three different positions

	Wavelength Shift (nm)		
	Position I (green) 3/4 of arm length	Position II (blue) 1/2 of arm length	Position III (gray) 1/4 of arm length
Actuated-no object	0.3025	0.1663	0.0345
Actuated-15g sphere	0.3262	0.2287	0.0459
Actuated-25g sphere	0.3297	0.2095	0.0635
Actuated-35g sphere	0.3449	0.2211	0.0802
Deactuated	0	0	0

**Table 2:** Wavelength shift of FBG with different sinusoidal embedding period.

	Wavelength Shift (nm)		
	Period I (green) ω = 0.5	Period II (blue) ω = 1	Period III (gray) ω = 2
Actuated-no object	0.1472	0.0382	0.0207
Actuated-15g sphere	0.1661	0.0507	0.0276
Actuated-25g sphere	0.1747	0.0627	0.0352
Actuated-35g sphere	0.1825	0.0822	0.0394
Deactuated	0	0	0

**Table 3:** Power change (comparing with natural bending value) observation under various movements and status change of the soft gripper.

Event	Condition	Power Change Magnitude	Power Change Pattern
Actuation	Holding an object	Large (>1.5 mW)	Instantaneously
	Failed to hold an object	Small (<0.5 mW)	Instantaneously
	Fail to actuate (air is leaking)	No change	No change
External perturbation	Weak	Large, with small fluctuation (+/−1 mW)	Fluctuate within a small power range then stabilize at a similar power as before perturbation
	Object position is changed	Small, with large fluctuation (+/−2 mW)	Fluctuate within a large power range then stabilized at a smaller power change value
	Object is lost	Small	Power return to a value that is close to natural bending position
Deactuation	Deflated	No change	Slightly overshoot and stabilized at natural bending value

**Table 4:** Properties of soft robotic gripper with sinusoidal and straight FBG configurations

	Sinusoidal	Straight
Sensitivity	0.14 mW/g	0.02 mW/g
Stretchable	Yes	No
Delamination	No	Significant
Repeatability	Good (>50 times)	Poor
Response time	<0.5 s	0.5–2.5 s
Stabilization time	Instantaneous	>60 s
